# Early identification of acute kidney injury in the ICU with real-time urine output monitoring: a clinical investigation

**DOI:** 10.1186/s12882-021-02485-w

**Published:** 2021-08-26

**Authors:** Dafna Willner, Aliza Goldman, Hagar Azran, Tal Stern, Dvora Kirshenbom, Guy Rosenthal

**Affiliations:** 1 Hadassah Medical Center, Jerusalem, Israel; 2 RenalSense Ltd., Hamarpe 3, Jerusalem, Israel- Clinical Research Department, 3 Hamarpe St, Har Hotzvim, Jerusalem, Israel

**Keywords:** Acute kidney injury, KDIGO criteria, Oliguria, Urine output, Electronic monitoring, Serum creatinine, Length of stay

## Abstract

**Background:**

KDIGO (Kidney Disease: Improving Global Outcomes) provides two sets of criteria to identify and classify acute kidney injury (AKI): serum creatinine (SCr) and urine output (UO). Inconsistencies in the application of KDIGO UO criteria, as well as collecting and classifying UO data, have prevented an accurate assessment of the role this easily available biomarker can play in the early identification of AKI.

**Study goal:**

To assess and compare the performance of the two KDIGO criteria (SCr and UO) for identification of AKI in the intensive care unit (ICU) by comparing the standard SCr criteria to consistent, real-time, consecutive, electronic urine output measurements.

**Methods:**

Ninety five catheterized patients in the General ICU (GICU) of Hadassah Medical Center, Israel, were connected to the RenalSense*™* Clarity RMS*™* device to automatically monitor UO electronically (UO_elec_). UO_elec_ and SCr were recorded for 24–48 h and up to 1 week, respectively, after ICU admission.

**Results:**

Real-time consecutive UO measurements identified significantly more AKI patients than SCr in the patient population, 57.9% (*N* = 55) versus 26.4% (*N* = 25), respectively (*P* < 0.0001). In 20 patients that had AKI according to both criteria, time to AKI identification was significantly earlier using the UO_elec_ criteria as compared to the SCr criteria (*P* < 0.0001). Among this population, the median (interquartile range (IQR)) identification time of AKI UO_elec_ was 12.75 (8.75, 26.25) hours from ICU admission versus 39.06 (25.8, 108.64) hours for AKI SCr.

**Conclusion:**

Application of KDIGO criteria for AKI using continuous electronic monitoring of UO identifies more AKI patients, and identifies them earlier, than using the SCr criteria alone. This can enable the clinician to set protocol goals for earlier intervention for the prevention or treatment of AKI.

## Background

Studies using the KDIGO (Kidney Disease: Improving Global Outcomes) criteria have identified acute kidney injury (AKI) in up to 75% of critically ill hospitalized patients [1]. The KDIGO criteria classify AKI based on: a rise in serum creatinine (SCr), a decrease in urine output (UO) over time, or both [2–4].

In the intensive care unit (ICU) neither of these indicators for AKI provides timely information about kidney injury. They are dependent on the times when SCr or UO is manually measured and recorded by the medical staff. Furthermore, SCr measurements are affected by many individual, potentially confounding, factors of the hospitalized patient. Most importantly, SCr levels increase only after approximately 50% loss of renal function, and is thus recognized as a late indicator for kidney injury [5–7].

As for UO, KDIGO defines oliguria as a urine output of less than 0.5 ml/kg. AKI severity (stages) is established according to their guidelines for extended periods of oliguria (beyond 6 h, e.g., stage 1 for 6 h of oliguria). Unfortunately, the application of the KDIGIO UO criteria has been inconsistent [1–4]. Additionally, a recent review comparing KDIGO criteria to its predecessors (RIFLE (Risk, Injury, Failure, Loss and End stage disease) and AKIN (Acute Kidney Injury Network)), has shown that the reported incidence of AKI varies depending on how the criteria are applied [8].

There have been few prospective studies applying the AKI UO criteria, and even fewer have incorporated *both* SCr and UO criteria. The studies also vary in how they record and assess UO. Some studies have applied the UO criteria as an average UO over 6, 12, and 24 h intervals, using either blocks or continuous windows. Others have interpolated data by averaging UO over the missing hours in nursing records. Oftentimes, there is no indication at all of how UO measurements used in the study were recorded [1, 7, 9–11]. This lack of uniformity in measurement, recording, application and reporting of UO makes it difficult to draw consistent conclusions from these studies.

It is not surprising, therefore, that the vast majority of retrospective studies to explore AKI using severity scores have applied only the SCr criteria. The few studies to include UO alongside SCr have either used 24-h averages, applied UO criteria only when available, or simply excluded patients from the study group when there was no UO measurement available [8, 11–14]. The resultant inconsistencies in classifying AKI according to UO have hindered the use of this easily available biomarker for early identification of AKI.

To address this gap in the research, a prospective study was designed using a novel, real-time UO measurement tool to consistently apply the UO criteria for AKI as defined in the KDIGO guidelines [15]. The UO criteria were also compared to the more commonly-applied SCr criteria, both being used to identify AKI in the ICU patient.

### Study goal

To assess and compare the performance of the two KDIGO criteria (SCr and UO) for identification of AKI in the ICU by comparing the standard SCr criteria to real-time, consecutive electronic urine output measurements.

## Methods

### Study design

Ninety five catheterized patients hospitalized in the General ICU (GICU) at Hadassah Medical Center, Jerusalem, Israel, were enrolled in a pilot study between August 2015 and November 2018. Local internal review board (IRB) approval was obtained and informed consent was waived.

#### Inclusion criteria

Patients > 18 years of age, SCr baseline within normal range prior to commencement of UO_elec_ observations, or stable SCr measurements compared to 1 month prior to ICU admission in patients with diagnosed chronic kidney injury.

#### Exclusion criteria

Patients isolated with bacterial infections such as methicillin-resistant *Staphylococcus aureus* (MRSA), vancomycin-resistant enterococcus (VRE), and Klebsiella; patients likely to be discharged or die within 24 h in the ICU; patients on dialysis; and pregnant women.

### Patient characteristics

Patient information included age, weight, baseline serum creatinine and daily serum creatinine measurements, primary diagnosis, co-morbidities, the need for mechanical ventilation, and the use of vasoactive drugs were recorded for up to 7 days or until discharge from the ICU.

### The RenalSense clarity RMS device

**Fig. 1:**
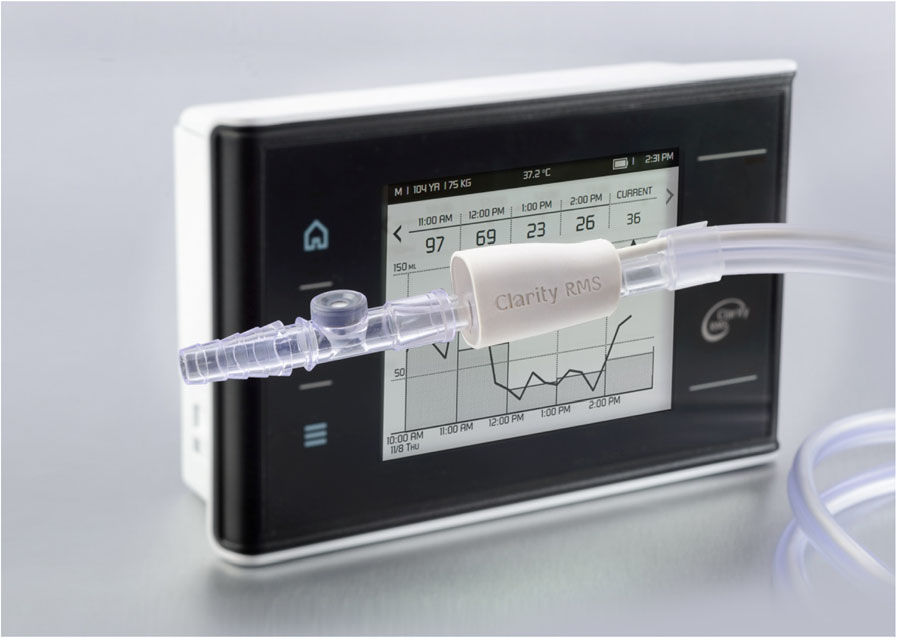
The RenalSense Clarity RMS Sensor Kit incorporates a proprietary sensor in a standard urinary drainage assembly to monitor urine output as it exits the Foley catheter. The data is communicated to the Clarity RMS Console through a cable integrated within the custom-designed drainage tube to the bag and then to the Console, mounted on the footboard of the patient bed

The RenalSense Clarity RMS device (Fig. 1) was used to electronically monitor UO every hour, and its technology is described elsewhere [15]. The data for the validation of the system was obtained from the first group of patients enrolled in this study. The electronic measurements were compared to manual UO measurements and the results were published previously. In brief, a total of 1046 h of electronic measurements recorded from 23 subjects measured with the RenalSense system were shown to be closer, with a better correlation, and narrower limits of agreement than the measurements obtained by the nurses, as compared to the scientific scale data [15].

For the purposes of this study the Clarity RMS Sensor Kit (a urine drainage apparatus incorporating a proprietary in-line sensor) was modified to incorporate a standard manual urinometer for the nursing staff to record UO as per their standard practice. Additionally, the sensor was connected by a cable to a RenalSense-designed data collection subsystem (DCS). Nursing staff was blinded to the Clarity RMS measurements console. No information recorded in the DCS had any bearing on the medical staff records, treatment interventions, or medical decisions for the patient. The drainage bag was placed in a container on a scientific scale (Precisa BJ 2200C) to be used as the reference for validation of the sensor measurements. The scale data were also recorded continuously by the same system.

### AKI analysis

AKI was identified and scored according to the KDIGO criteria for both SCr and UO [2]. KDIGO Stage 1 is an increase in SCr by ≥0.3 mg/dl (≥26.5 mol/l) within 48 h or an increase in SCr to 1.5–1.9 times baseline or urine volume < 0.5 ml/kg/h for at least 6 h and up to 12 h. Stage 2 is an increase in SCr to 2.0–2.9 times baseline or urine volume < 0.5 ml/kg/h for ≥12 h. Stage 3 is SCr > 3.0 times baseline or initiation of renal replacement therapy (RRT) or urine volume < 0.3 ml/kg/h for ≥24 h or anuria for ≥12 h [1, 2].

AKI SCr was assessed for up to 7 days when measurements were available, following KDIGO guidelines that allow for use of a rolling baseline in the 7 days after admission for diagnosis of AKI by SCr [1, 2]. More than 50% of patients had no electronic record of SCr prior to hospital entry. Therefore, a uniform baseline SCr was defined as the first SCr drawn upon ICU admission.

Patients diagnosed in their medical records with chronic kidney injury as identified by their SCr were included if there was no evidence of “acute on chronic” kidney injury (identified by an increase in SCr upon initial ICU admission compared to previous measurement, or physician report referencing an acute increase).

SCr measurements were collected twice daily by the nurses according to department protocol and patients were treated accordingly as per standard of care. SCr measurements for the study included twice-daily measurements for up to 7 days following Clarity RMS removal if they remained in the ICU. Patients were connected to the Clarity RMS sensor kit upon admission to the ICU for a minimum of 24 h. AKI staging per KDIGO was applied to patient UO monitored consecutively and electronically up to 48 h after admission to the ICU.

#### Identification of AKI and time to identification of AKI

AKI was diagnosed in the study group by two different methods corresponding to the two sets of KDIGO criteria (UO and SCr).

The first method to identify AKI was based on UO following 6 h of oliguria as defined by the first stage of the KDIGO criteria. Patients were divided into AKI UO_elec_ versus non AKI UO_elec_.

The second method to identify AKI was an increase of SCr in patient records as defined by the first stage of the KDIGO criteria. Patients were divided into AKI SCr versus non AKI SCr. We compared the identification according to each of these methods separately.

#### Length of stay (LOS) (and related factors)

For this analysis, the population was divided differently than for AKI identification. This was done to isolate the AKI patients identified by the UO_elec_ criteria alone (i.e., not by SCr criteria). The goal was to assess whether LOS or other related factors had a positive association with AKI UO_elec_ in the absence of AKI SCr.

Thus, for the LOS analysis, the patients were divided into 3 mutually exclusive groups:

- Group **AKI-SCr** for all of those patients classified as AKI by their SCr criteria and without regard to their UO_elec_ measurements (i.e., this group includes both those identified as AKI SCr alone and those identified by ACI SCr *and* AKI UO_elec)_.
- Group **AKI-UO**_**elec**_**′** for those patients classified as AKI using UO_elec_ criteria alone, i.e., not including those already in group **AKI-SCr**. This group (with the prime symbol) is a subset of the previously described AKI UO_elec_ group. This group represents those patients who *would not* have been identified as AKI using the standard SCr criteria.
- Group **non-AKI** for those patients who were not classified as suffering from AKI, according to either SCr or UO criteria.

LOS in the ICU and hospital, hospital readmissions within 3 months of ICU discharge, RRT, and all-cause mortality were recorded during the entire length of the study and up to a year after the study was completed.

### Statistical analysis

Baseline characteristics for the study population are presented. For continuous variables, mean, standard deviation (SD), median and IQR are presented. For dichotomous variables, count and proportion are presented. Rate of AKI is described according to various identification groups. A comparison between the rate of UO_elec_ identification and SCr identification was performed using Wald test.

For all patients with AKI, time to identification is presented using Kaplan-Meier curves. Comparison between the different measures’ identification times was performed using the log-rank test. This analysis was repeated for the patients identified by both UO_elec_ and SCr. Comparison between AKI identification groups was performed using the Wilcoxon rank sum test. LOS in the ICU and general hospitalization are presented by mean, SD, median, IQR, and range.

A comparison between all groups was performed using Kruskal-Wallis test, as well as specific post hoc between-group comparisons, using Wilcoxon rank sum test.

In order to account for mortality as censored data, sensitivity analyses were performed analyzing these parameters as time to discharge, using Kaplan-Meier curves and the log-rank test. These analyses were repeated for subpopulations by age. No adjustment for multiple comparisons was performed. All analyses were performed using R 3.6.2.

## Results

### Patient characteristics

The patient population was 67% male and 37% of the population was over 70 years of age with a median age of 63. As expected, in the GICU, the majority of the patients in our study group were surgical (67%). Patient characteristics including comorbidities and AKI risk factors are shown in Table 1.

**Table 1:** Patient characteristics

patient information ***N*** = 95		
**gender-** **% (n)**	**male**	67% (64)
	**female**	33% (32)
**age**	**mean (SD)**	59.3 (19.7)
	**median (IQR)**	63 (47.5, 74)
**weight**	**mean (SD)**	79.8 (16.6)
	**median (IQR)**	75 (70, 90)
**cause for admission-** **% (n)**	**surgical**	66% (63)
	**oncological**	12% (11)
	**burns**	4% (4)
	**trauma**	32% (30)
	**neurological**	21% (16)
	**infection/sepsis**	11% (10)
	**cardiological**	0% (0)
	**other**	14% (13)
**risk factors for AKI-** **% (n)**	**age > 70**	37% (35)
	**diabetes mellitus**	24% (23)
	**hypertension**	29% (28)
	**morbid obesity**	7% (7)
	**chronic liver disease**	7% (7)
	**congestive heart failure**	7% (7)
	**chronic lung disease**	12% (11)
	**Ischemic heart disease**	12% (11)
	**mechanical ventilation**	76% (72)
	**ianotropes**	44% (42)
	**chronic kidney disease**	1% (1)

### Identification of AKI

**Fig. 2:**
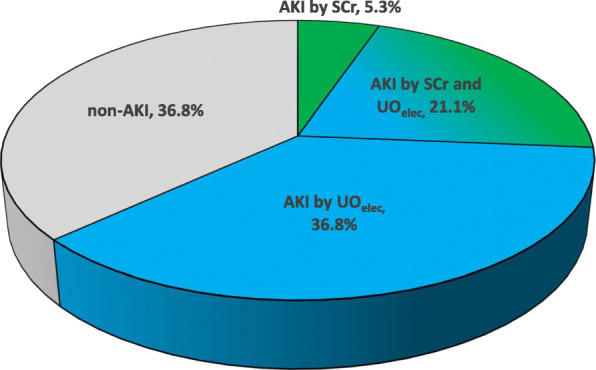
Patients in the study population with, or without, AKI classified according to the KDIGO guidelines by UO_elec_, SCr, or both criteria

**Fig. 3:**
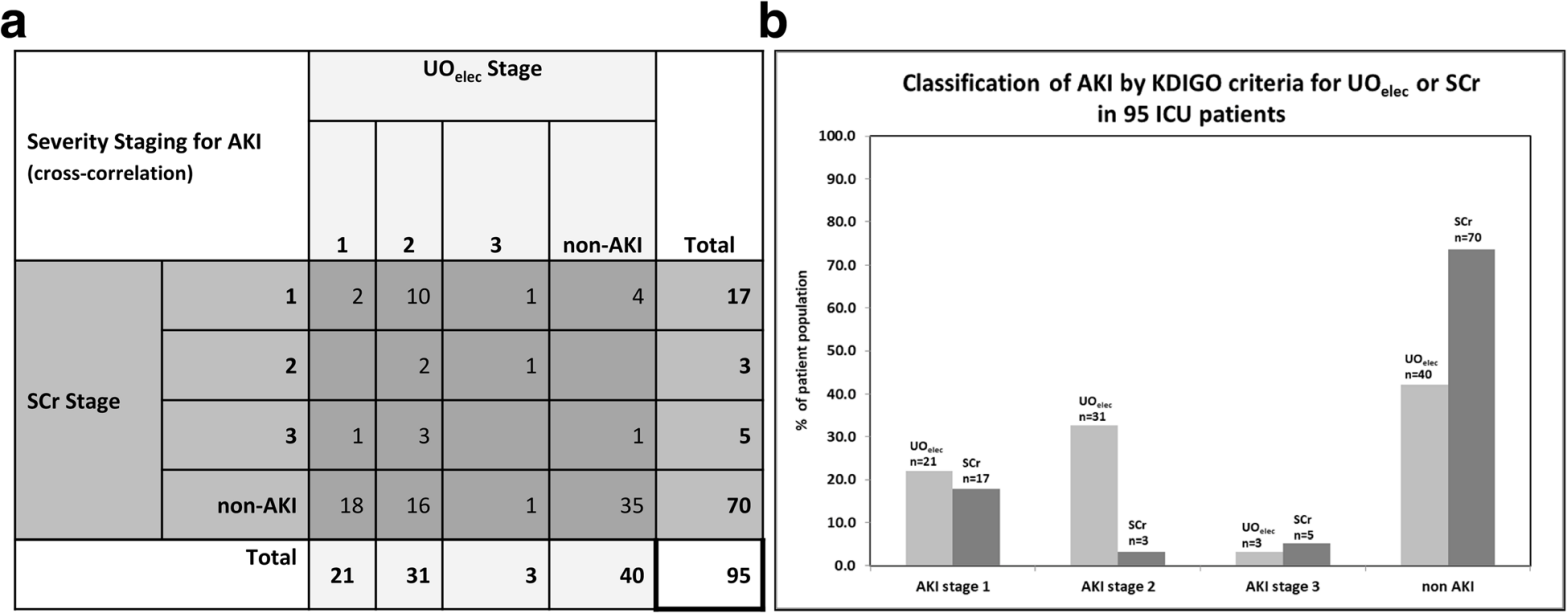
**a** AKI severity staging in the study population according to the KDIGO guidelines, highlighting the cross correlation between the patient stages according to UO_elec_ and SCr criteria. **b** Comparison of patients classified in each severity stage of AKI for UO_elec_ versus SCr KDIGO criteria

A total of 60 out of the 95 (63.2%) patients in the study group were identified with AKI, applying the KDIGO criteria, using either SCr or UO_elec_ or both criteria (Fig. 2). Twenty patients had AKI according to both UO_elec_ and SCr criteria, while 5 were identified only by SCr criteria and 35 were identified only by UO_elec_ measurements (Fig. 3a).

In total, AKI UO_elec_ was identified in 57.9% (*N* = 55) of the patient population and AKI SCr was identified in 26.4% (*N* = 25) of the patient population. Out of the 60 AKI patients defined by the KDIGO classification, UO_elec_ identified significantly more patients than SCr, 92% versus 42%, respectively, *P* < 0.0001 (Figs. 2, and 3b).

### AKI severity

Out of the 55 AKI UO_elec_ patients, 38% reached a maximum KDIGO score of stage 1, and 62% reached a maximum severity of stage 2 or 3. Out of the 25 patients with AKI SCr, 68% of the patients had a maximum severity score of stage 1 and 32% reached stages 2 or 3 as their maximum KDIGO severity score (Fig. 3a and b).

### Time to identification of AKI

**Fig. 4:**
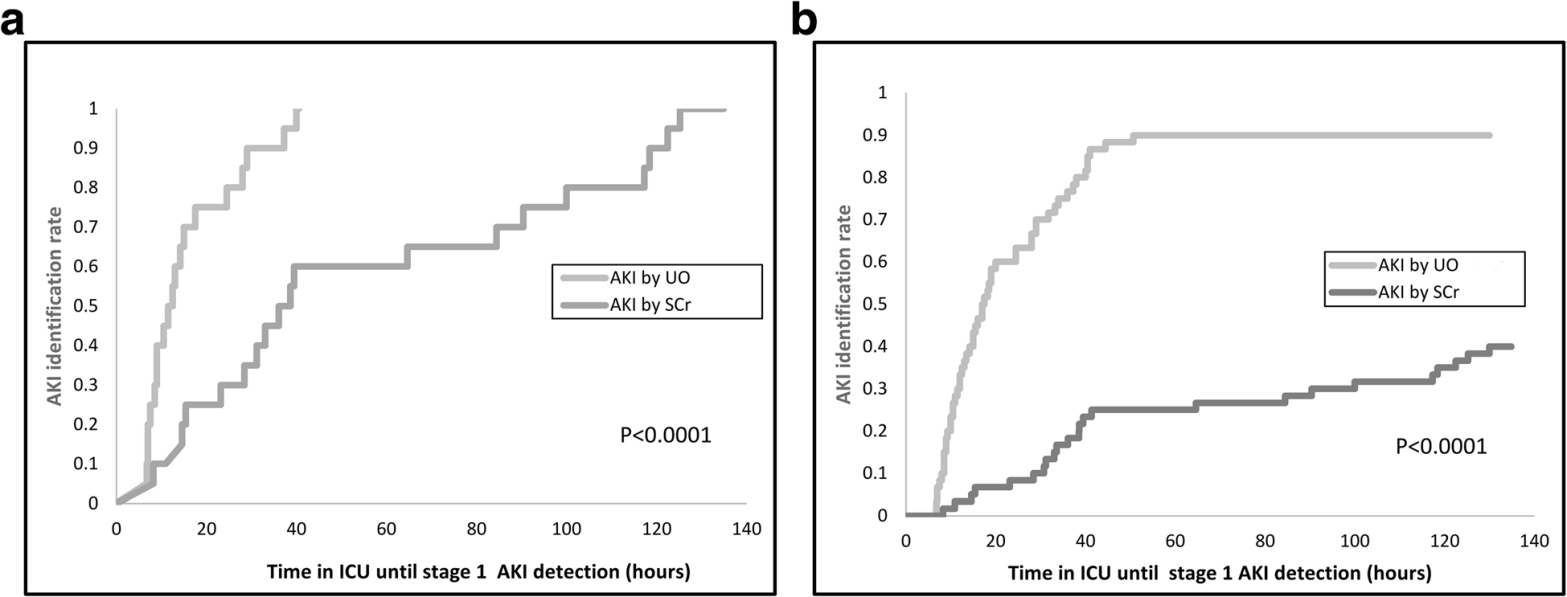
**a** Kaplan-Meier curve for time to identification rates of AKI for the 60 patients identified applying either UO_elec_ or SCR criteria. **b** Kaplan-Meier curve for time to identification rates of AKI for the 20 patients identified applying both SCr and UO_elec_ criteria

**Fig. 5:**
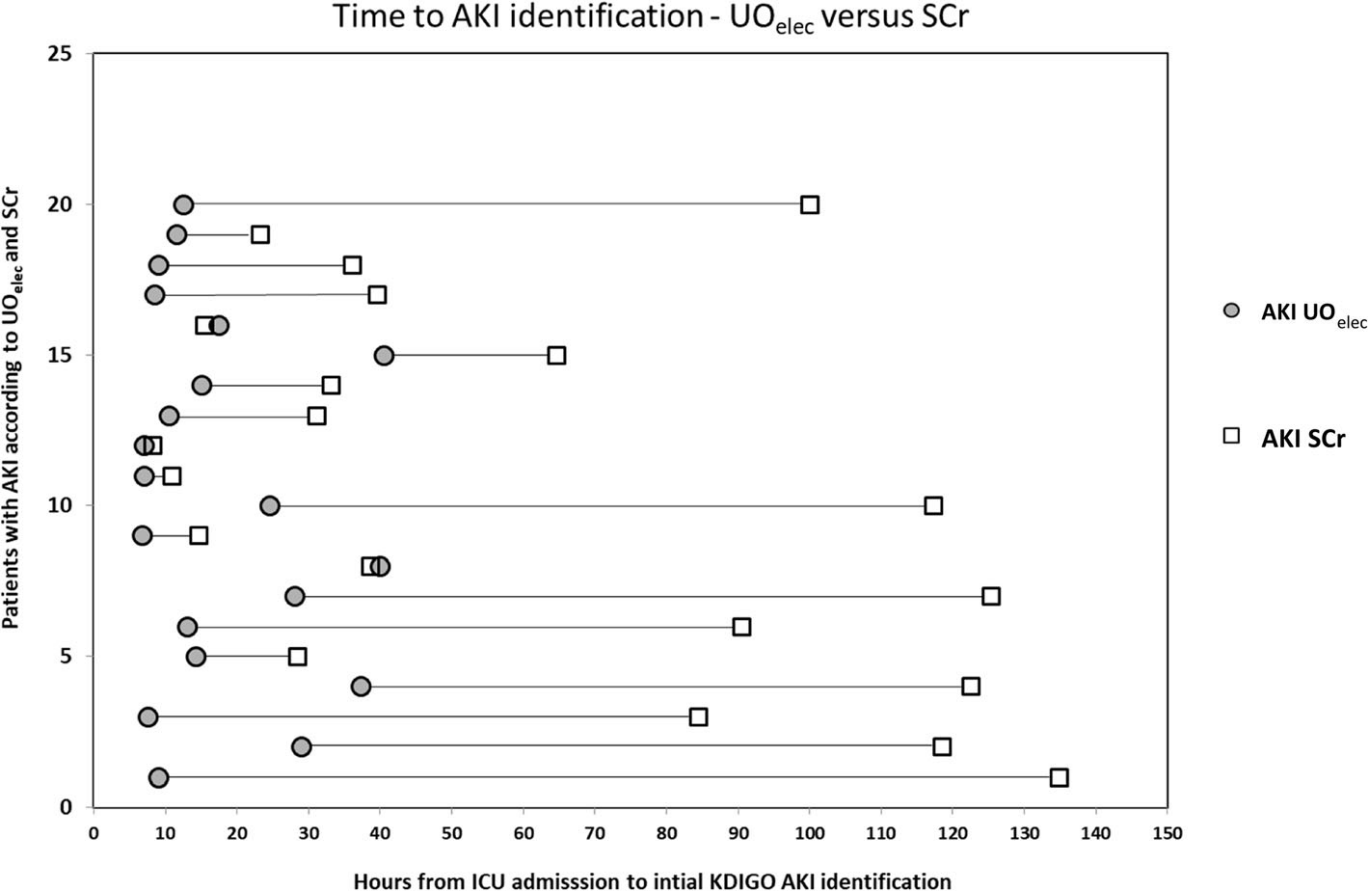
Time from admission to the ICU until AKI identification for the 20 patients who had AKI according to both UO_elec_ and SCr criteria. Time of identification is shown for each of the criteria for each patient

Out of the 60 AKI patients identified by at least one of SCr or UO_elec_, the time to identify AKI using UO_elec_ was significantly earlier than with SCr (*P* < 0.0001) (Fig. 4a). Moreover, in the 20 patients that had AKI according to both criteria, time to AKI identification was significantly earlier using UO_elec_ as compared to SCr (*P* < 0.0001). Among this population, the median (IQR) identification time was 12.75 (8.75, 26.25) hours for AKI UO_elec_ and 39.06 (25.8, 108.64) hours for AKI SCr (Figs. 4b, and 5).

### Length of stay and related factors

#### LOS in the ICU

There was no significant difference in the LOS in the ICU for the AKI-UO_elec_′ group as compared to the non-AKI group (*P* = 0.3354). LOS in the ICU for the AKI-SCr group was significantly longer than the AKI-UO_elec_′ group (*P* = 0.0043) (Table 2).

**Table 2:** Time to discharge

				AKI-SCr				AKI-UO_**elec**_′				non-AKI			Overall *P*-Value	*P*-value For specific categories	
	Hospitalization	**LOS in**	**N**	**Mean (SD)**	**Median (IQR)**	**Range**	**N**	**Mean (SD)**	**Median (IQR)**	**Range**	**N**	**Mean (SD)**	**Median (IQR)**	**Range**		**SCr vs UO**	**UO vs Non AKI**
**All patients**	Initial admission (days)	ICU	**25**	14.7 (17.91)	7.98 (4.28, 18.72)	(2.04, 86.79)	**35**	6.59 (6.39)	3.09 (2.06, 8.02)	(1.09, 22.97)	**35**	5.17 (5.03)	3.08 (1.86, 7.23)	(1.09, 24.38)	0.0230	0.0043	0.3354
		Hospital	**25**	28.71 (27.44)	20.39 (8.48, 39.46)	(2.04, 118.21)	**35**	28.79 (26.75)	20.27 (10.08, 45.99)	(2.42, 101.99)	**35**	21.34 (17.6)	14.92 (8.88, 30.46)	(1.61, 72.46)	0.8184	0.9343	0.4210
	Total hospitalization days in 3 months		**25**	32.63 (28.62)	23.42 (9.7, 55.56)	(2.04, 118.21)	**35**	32.93 (29.08)	22.04 (10.08, 56.08)	(2.42, 104.99)	**35**	23.09 (18.22)	16.23 (9.4, 38.15)	(1.61, 72.46)	0.7787	0.8867	0.3296
**Age ≤ 70**	Initial Admission (days)	ICU	**12**	10.86 (8.98)	7.92 (4.7, 11.35)	(3.84, 30.63)	**21**	6.32 (6.25)	3.09 (2.72, 7.53)	(1.09, 22.97)	**27**	5.58 (4.93)	3.96 (1.93, 7.45)	(1.09, 24.38)	0.0058	0.0184	0.9255
		Hospital	**12**	27.45 (17.91)	23.84 (13.82, 38.72)	(5.82, 59.86)	**21**	33.31 (30.28)	28.42 (8.46, 48.18)	(2.52, 101.99)	**27**	23.81 (17.12)	16.31 (10.5, 31.25)	(1.93, 72.46)	0.1902	0.9403	0.655
	Total hospitalization days in 3 months		**12**	31.45 (21.64)	28.79 (15.37, 47.72)	(5.82, 70.42)	**21**	38.12 (32.47)	34.44 (8.46, 56.48)	(2.52, 104.99)	**27**	26.07 (17.59)	18 (12.54, 40.25)	(6.48, 72.46)	0.1240	0.9701	0.5262
**Age > 70**	Initial Admission (days)	ICU	**13**	18.26 (23.22)	9.7 (3.52, 23.15)	(2.04, 86.79)	**14**	7.00 (6.81)	3.68 (1.93, 13.27)	(1.69, 22.04)	**8**	3.8 (5.49)	1.83 (1.57, 2.54)	(1.21, 17.33)	0.0008	0.0584	0.0654
		Hospital	**13**	29.88 (34.78)	18.72 (6.49, 41.04)	(2.04, 118.21)	**14**	22.01 (19.43)	13.31 (11.29, 22.35)	(2.42, 65.29)	**8**	13.02 (17.71)	6.81 (3.7, 13.1)	(1.61, 55.33)	0.6549	0.8842	0.0653
	Total hospitalization days in 3 months		**13**	33.73 (34.74)	20.39 (8.48, 55.56)	(2.04, 118.21)	**14**	25.15 (21.92)	14.88 (11.29, 36.35)	(2.42, 65.99)	**8**	13.02 (17.71)	6.81 (3.7, 13.1)	(1.61, 55.33)	0.5115	0.8843	0.0478

Comparison of time to discharge from the ICU and the hospital, and readmissions to the hospital within 3 months in patients with AKI according SCr criteria (AKI-SCr), according to UO only (AKI-UO_elec_′), and patients that did not have AKI by either criteria (non-AKI). The groups were also divided and compared according to the cut-off of above and below 70 years old

#### LOS in the hospital

the AKI-SCr group had a median (IQR) LOS in the hospital of 20.39 (8.48, 39.46) days. The AKI-UO_elec_′ group had a similar LOS in the hospital with a median of 20.27 (10.08, 45.99) days. Although it did not reach significance, this was approximately 5 days longer than the non-AKI group, 14.92 (8.88, 30.46) days (*P* = 0.4210) (Table 2).

#### LOS in the hospital with a cut-off below and above 70 years of age

Patients less than 70 years old in the AKI-UOelec′ group had the longest median (IQR) hospital stay, 28.42 (8.46, 48.18) days, as compared to the AKI-SCr group, and the non-AKI group of 23.84 (13.82, 38.72), and 16.31(10.5, 31.25) days, respectively (*P* = 0.9403 and *P* = 0.655, respectively) (Table 2).

Sensitivity analyses for these above parameters as time to discharge (taking into account censored data due to mortality) yielded similar results.

## Discussion

The RIFLE, AKIN, and KDIGO criteria for AKI have recognized the need to incorporate smaller time intervals for the assessment of kidney injury using UO [2, 16]. A recent study in ICU and cardiac surgery patients compared consecutive hourly measurements of UO to mean UO over the time measured. In the ICU patients it was shown that using a mean UO, rather than consecutively measured hours, led to misclassification of the KDIGO severity stage. For cardiac surgery patients the study found that averaging UO over time, versus consecutive hourly measurements, significantly overestimated the incidence of AKI [9].

Detection of oliguria is limited by the available tools, human error, and varying approaches to its definition. A reliable method for consecutive measurements of UO would provide a consistent application of the KDIGO UO criteria for diagnosis of AKI. In this study we demonstrated that consecutive electronically monitored UO identified significantly more cases of AKI according to the KDIGO criteria that were not identified by SCr alone. Additionally, in patients that fulfilled AKI definitions using both the SCr and UO criteria, UO_elec_ measurement identified AKI significantly earlier than SCr.

The advantage of continuous measurements of UO was presented in a study showing oliguric periods as a predictor of higher mortality in critically ill patients. The authors discussed the need for a broader understanding of UO as a continuous physiological variable as opposed to one measured in set intervals [17].

It has also been argued that UO is too sensitive a biomarker for identifying AKI since oliguria may occur in response to normal physiological mechanisms [16, 18–20]. However, many studies confirm that oliguria, even in the absence of a rise in creatinine, identifies patients who have worse short-term and long-term outcomes, increased LOS, increased severity of AKI, and require more dialysis [1, 3, 4, 13, 17].

Our study was blinded, and UO for the patients was not documented in their records according to the electronic monitoring. The course of patient treatment was not based upon the consecutive electronic monitoring of UO, so the electronic monitoring did not impact ICU outcomes. This may explain why the patients with AKI as defined by UO_elec_ had a similar LOS to that of the non-AKI population. However, the extended time in the hospital after ICU discharge for the patients with AKI as defined by UO_elec_ (> 5 days in the general population and 8 days in the under-70 group Table 2) compared to the non-AKI patients, suggest there may be clinical implications for earlier identification of AKI using UO_elec_.

Retrospective studies have shown a prolonged LOS and higher mortality in the ICU and cardiac surgery patients that had AKI according to both the UO and SCr criteria [8, 21]. Physicians in our study were not blinded to SCr measurements (as they were with the UO_elec_ measurements), which may have contributed to the LOS in the ICU observed in the AKI-SCr group in our study. This may be further highlighted with the similar overall LOS in the hospital in the AKI-SCr (20.39 days) and the AKI-UOelec′ (20.27 day) groups, despite the significant difference between these groups for their median LOS while in the ICU.

A retrospective study in over 15,000 adults compared intensive vs non-intensive monitoring of UO and intensive vs non-intensive SCr monitoring. Intensive UO monitoring was shown to be independently associated with improved survival to 30 days among patients developing AKI. Intensive monitoring of SCr had no effect on 30-day mortality associated with AKI. With or without AKI, patients who had intensive UO monitoring had significantly less cumulative fluid volume and fluid overload. They were also significantly less likely to receive vasopressors over the first 72 h of their ICU stay [22]. Intensive monitoring in that study was considered a measurement recorded at least once every 3 h and was exclusively manual monitoring of UO. Our study shows that automated, continuous, consecutive hourly monitoring of UO can provide a uniform application of KDIGO criteria to identify AKI early and in real-time. This may provide support to improve outcomes similar to that found in intensive manual monitoring, and merits further study. Furthermore, a uniform and consistent application of UO data for early identification of AKI may enable a consensus definition for, and earlier intervention to prevent progression of the disease [17].

### Study limitations

In our study, UO_elec_ measurements were recorded every hour and SCr measurements were taken twice a day (which is considered very frequent for standard of care [22]). This gives UO_elec_ a certain advantage in identifying AKI by assessing the previous 6 hours once an hour, while SCR can only identify AKI approximately once in 12 h. This inherent limitation also highlights an inherent advantage of the use of UO_elec_ criteria for the early identification of AKI. This is unlikely to change due to the labor-intensive and invasive nature of SCr tests.

Due to the nature of the pilot study, our patient population was limited in size. Follow-up for readmissions within 3 months after hospital discharge and 1 year mortality, as well as time to RRT were too small for analysis. Further prospective studies using real-time continuous UO_elec_ monitoring on larger patient cohorts that includes tracking fluid balance, and fluid response to diuretics should be designed to assess the advantage of early detection of AKI and these additional criteria.

## Conclusion

Electronic UO monitoring provides a uniform and consistent definition for identifying AKI patients in the ICU according to the KDIGO UO criteria. Application of KDIGO criteria for AKI using continuous monitoring of UO identifies more AKI patients, and identifies them earlier, than using the SCr criteria alone. This tool can enable the clinician to set protocol goals for earlier intervention for the prevention or treatment of AKI.

## Data Availability

The datasets used and/or analyzed during the current study are available from the corresponding author on reasonable request.
